# Intravenous Administration of Cisplatin with Magnesium Sulfate Supplement May Prevent Kidney Toxicity in Rats: The Role of Gender and Magnesium Sulfate Dose

**DOI:** 10.1155/2022/1218222

**Published:** 2022-02-16

**Authors:** Parisa Pournaderi, Behnaz Pourvali, Farzaneh Ashrafi, Ardeshir Talebi, Zahra Pezeshki, Mehdi Nematbakhsh

**Affiliations:** ^1^Water & Electrolytes Research Center, Isfahan University of Medical Sciences, Isfahan, Iran; ^2^Department of Internal Medicine, Oncology-Hematology Section, Isfahan University of Medical Sciences, Isfahan, Iran; ^3^Department of Clinical Pathology, Isfahan University of Medical Sciences, Isfahan, Iran; ^4^Department of Physiology, Isfahan University of Medical Sciences, Isfahan, Iran; ^5^Isfahan MN Institute of Basic & Applied Sciences Research, Isfahan, Iran

## Abstract

**Background:**

Cisplatin (CP) is widely used to treat various kinds of malignancies, but to avoid its side effects of nephrotoxicity and hypomagnesemia, magnesium supplementation is a subject of debate. The current study was designed to determine the protective role of intravenous magnesium sulfate (MgSO_4_) against intravenous administration of CP in male and female rats.

**Method:**

In this case-control experimental study, 80 Wistar male and female rats in 12 groups of experiments were subjected to receive intravenous administration of CP accompanied with intravenous infusion of different doses (1, 3, and 10 mg/ml solution) of MgSO_4_ and were compared with the control groups.

**Results:**

CP administration increased blood urea nitrogen (BUN), creatinine (Cr), kidney tissue damage score (KTDS), and kidney weight (KW), and they were attenuated by the mid-dose of MgSO_4_ supplementation in female rats. However, in male rats, the increase of Cr, BUN, KTDS, and KW induced by CP was ameliorated by low, mid-, and high doses of MgSO_4_ supplements. The levels of these markers were significantly different between male and female rats in the mid-dose of MgSO_4_-treated group (BUN: *P*=0.002, Cr: *P*=0.005, KTDS: *P*=0.002, and KW: *P*=0.031). CP reduced clearance of Cr (ClCr) in both male and female rats significantly compared to the control group of saline alone (*P*_male_ = 0.002 and *P*_female_ = 0.001), and the mid- and high doses of MgSO_4_ supplements improved ClCr in female rats. There were also sex differences in ClCr in mid- (*P*=0.05) and high (*P*=0.032) doses of MgSO_4_-treated groups. CP accompanied with the mid-dose of MgSO_4_ supplement reduced the KTDS (*P*_male_ = 0.04 and *P*_female_ = 0.004) and KW (*P*_male_ = 0.002 and *P*_female_ = 0.042) in both male and female rats significantly when compared with the CP-alone-treated group, while there were also significant differences between the sexes (KTDS: *P*=0.002 and KW: *P*=0.031). CP accompanied with three different doses of MgSO_4_ supplements did not improve the serum levels of lactate dehydrogenase, urine level of sodium, malondialdehyde, urine flow, and nitrite statistically when compared with the CP-alone-treated group.

**Conclusion:**

The renal protective effect of MgSO_4_ could be dose and gender related.

## 1. Introduction

*cis*-Diamminedichloroplatinum, abbreviated cisplatin (CP), is a platinum-based chemotherapeutic compound widely used to treat various kinds of malignancies including testicular, ovarian, uterine, cervical, breast, head and neck, bladder, stomach, esophageal, and lung cancers [1–5]. CP was synthesized in 1844, and its chemical structure was first identified in 1893 [6], and the idea for chemotherapy was formed [7]. Finally, it became available for oncology treatment in 1978 [8].

Renal tubular dysfunction and acute and chronic renal failures are the most important side effects of CP that occur in 15 to 30% of patients [9, 10]. More than 50% of CP is excreted through urine by the first day of drug administration, and the CP concentration in kidney cells is several folds higher than the other organs [11]. In fact, CP accumulates primarily in the S3 segment of the proximal tubule, and accumulation of CP in the proximal tubule frequently leads to dose-limiting CP-induced nephrotoxicity (CPIN).

Kidney toxicity induced by CP also occurs by the production of oxygen free radicals (ROS), especially hydroxyl radicals, which cause peroxidation of fats, oxidation of proteins and nucleic acids, and destruction of cell membranes, and these radicals reduce glomerular filtration rate (GFR) and cause acute nephrotoxicity [12]. The kidneys are able to produce a low amount of ROS in the physiological condition during metabolism, but in disease conditions and by injured cells due to the imbalance between the pro-oxidant and antioxidant, the activity of mitochondrial scavengers alters, and the ROS production increases and promotes renal injury [12].

Hypomagnesemia is a well-known complication of CP therapy that is seen in more than half of the CP-treated patients. Hypokalemia is another complication of CP therapy that may be indirect and secondary to CP-induced hypomagnesemia. Therefore, in the hydration protocol, usually, two grams of magnesium sulfate (MgSO_4_) and 20 milliequivalents of potassium chloride (KCl) add to each liter of isotonic saline to prevent hypokalemia and hypomagnesemia and to avoid the side effects of CP [13]. Magnesium (Mg) itself has an antioxidant effect against the ROS production. The Mg deficiency promotes oxidative stress and apoptosis [14, 15]. Mg also affects in other organs such as the heart [16], liver [14, 17], central nervous system [18], and gastrointestinal tract [19]. In chronic kidney disease and end-stage renal disease, the kidneys are not able to regulate Mg concentration [20], while Mg may have a protective role against chronic kidney disease [21].

GFR and plasma Mg concentration decrease after administration of CP at dose greater than 50 mg/m^2^, while high plasma concentration of free platinum is also directly associated with renal toxicity [22]. In diabetic CP-treated rats, Mg administration improved kidney tissue damage [23]. However, the intraperitoneal injection of MgSO_4_ was examined in rats treated with CP, and no significant protective effect was detected [24], while Willox and Zarif showed that Mg administration protects the kidneys against CPIN in patients [25]. CPIN also is age and gender related in an experimental study [26]. Despite all the studies, the role of MgSO_4_ supplementation remains uncertain and raises some questions in the clinic related to the effective dose of MgSO_4_ and the role of gender. In basic studies, CP usually is evaluated by peritoneal injection than by intravenous injection, as is done in the clinic. However, in order to have an experimental model similar to what happens in the clinic, in the current study, the protective role of intravenous MgSO_4_ injection against intravenous administration of CP was investigated.

On the contrary, different studies had been performed in two genders, and concomitant use of CP with antioxidants and other drugs showed different responses in males and females [27–29]. It is also shown that Mg at different doses has a dose-dependent effect on reducing renal toxicity [30–32]. As a result, due to hypomagnesemia that occurs during CP administration, it is necessary to investigate the effect of different doses of MgSO_4_ on renal toxicity caused by CP in both sexes. In order to obtain this objective, CP accompanied with hydration protocols containing different doses of MgSO_4_ was administered intravenously, and the results were evaluated.

## 2. Materials and Methods

Randomly, 80 Wistar rats including male (200–300 g) and female (140–200 g) were selected after adaptation to the laboratory environment with a temperature range of 23–25°C and 12 h light/12 h dark cycle, without any restrictions on water and rat chow. All the experimental procedures were approved in advance by the Isfahan University of Medical Sciences Ethics Committee (Code no. IR.MUI.MED.REC.1399.1176).

### 2.1. Hydration Protocol

Usually in the clinic, for a 70 kg patient, a total of 2 liters of fluid is considered as hydration volume before and after CP therapy. Accordingly, in a 200 g animal, the volume required for hydration will be about 5.5 ml. This volume of fluid was divided into 2 ml, 1.5 ml, and 2 ml for before, during, and after CP administration, respectively. In addition, the MgSO_4_ supplement in the clinic is about 2 mg/ml of hydration fluid, and based on this concentration, we assigned 3 doses of MgSO_4_ supplements as 1, 3, and 10 mg/ml [27].

The time to inject these volumes of fluid was assigned as 45, 30, and 45 minutes, respectively, using a microinfusion pump (New Era Pump Systems Inc., Farmingdale, NY, USA). These volumes of fluid contained the required supplements (Table 1).

### 2.2. Study Groups

We used 6 animals in each group calculated as described in the literature [33]. The control groups were assigned to receive either saline (vehicle) or saline accompanied with KCl and mid-dose of MgSO_4_ (3 mg/ml) in both sexes. The control groups did not receive CP. In addition, in order to determine the effect of CP, a group of animals was treated with CP alone in each sex. In order to determine the effect of the MgSO_4_ supplement against CPIN, we designed three groups of animals treated with CP and different doses of MgSO_4_. Accordingly, the details of 12 experimental groups are explained as follows:

  Groups 1 and 7 received saline continuously for a period of two hours (total injection volume was 5.5 ml). These groups were named “saline” groups (Table 1).
  Groups 2 and 8 received saline, KCl (0.015 meq/ml), and MgSO_4_ (3 mg/ml) continuously for a period of two hours (total infusion volume was 5.5 ml). These groups were named “saline + KCl + Mg3” groups (Table 1).
  Groups 3 and 9 received CP (7.5 mg/kg) (Malan Company; Athens, Greece) continuously for a period of half an hour (total infusion volume was 1.5 ml). These groups were named “CP” groups (Table 1).
  Groups 4 and 10 received CP (7.5 mg/kg) in saline, KCl (0.015 meq/ml), and MgSO_4_ (1 mg/ml) continuously for a period of half an hour (injection volume was 1.5 ml). However, before and after CP infusion, 2 ml of solution containing KCl (0.015 meq/ml) and MgSO_4_ (1 mg/ml) was administered. Therefore, the total infusion volume of fluid in each animal was 5.5 ml. These groups were named “CP + KCl + Mg1” groups (Table 1).
  Groups 5 and 11 received CP (7.5 mg/kg) in saline, KCl (0.015 meq/ml), and MgSO_4_ (3 mg/ml) continuously for a period of half an hour (infusion volume was 1.5 ml). However, before and after CP infusion, 2 ml of solution containing KCl (0.015 meq/ml) and MgSO_4_ (3 mg/ml) was administered. Therefore, the total injection volume of fluid in each animal was 5.5 ml. These groups were named “CP + KCl + Mg3” groups (Table 1).
  Groups 6 and 12 received CP (7.5 mg/kg) in saline, KCl (0.015 meq/ml), and MgSO_4_ (10 mg/ml) continuously for a period of half an hour (infusion volume was 1.5 ml). However, before and after CP infusion, 2 ml of solution containing KCl (0.015 meq/ml) and MgSO_4_ (10 mg/ml) was administered. Therefore, the total injection volume of fluid in each animal was 5.5 ml. These groups were named “CP + KCl + Mg10” groups (Table 1).

### 2.3. Surgical and Experimental Procedures

At the beginning of the experiment, rats were anesthetized with chloral hydrate (450 mg/kg, i.p., Merck, Germany) and xylazine (10 mg/kg, i.p.). To facilitate ventilation, endotracheal intubation was down. Femoral vein catheterization for drug and fluid infusions by the microinfusion pump according to the prescribed protocol (Table 1) was performed. At the end of the drug injection, the surgical site was sutured, and after regaining consciousness, the animals were transferred to the animal house, and they were observed for six days under standard conditions. The rats were weighed daily.

At the end of the sixth day, all the animals were placed in metabolic cages, and urine from each animal was collected for 4 hours, then the animals were anesthetized again, and blood samples were taken by heart puncture. After sacrificing, the kidneys were removed and weighted rapidly. Serum samples were obtained and stored in a freezer at −20°C for the measurement of biochemical factors, and the left kidney was placed in 10% formalin for histological investigation processes. The hematoxylin and eosin (H&E) staining was applied to examine the tubular damages. The tubular lesions were scored (by a pathologist who was blinded to the study protocol) from 1 to 4 (in the form of a parameter named kidney tissue damage score (KTDS)), while the score of zero was assigned to normal tubules without damage. The KTDS was given to each sample based on percentage intensity of hyaline cast, tubular atrophy, necrosis, vacuolization, and debris in the tubule. The normal tubule was considered as <5% damage. Grade 1 (5 < damage < 25%), grade 2 (25 < damage < 50%), grade 3 (50 < damage < 75%), and grade 4 (damage >75%) were assigned to evaluate the KTDS. The serum levels of creatinine (Cr), blood urea nitrogen (BUN), Mg, lactate dehydrogenase (LDH), and urine levels of sodium (Na) and Cr were measured using current laboratory methods. The serum levels of malondialdehyde (MDA) according to the manual methodology and nitrite by the Griess method were quantified. The clearance of Cr (ClCr) was calculated based on the renal clearance formula.

### 2.4. Statistical Analyses

Data were presented as mean ± SEM. One-way analysis of variance (ANOVA) followed by LSD as post hoc was applied to compare quantitative data between the groups, and the Student *t*-test was used to compare the data between sexes. The Kruskal–Wallis and Mann–Whitney *U* tests were employed to compare the KTDS between the groups. *P* values <0.05 were considered statistically significant.

## 3. Results

### 3.1. Serum Levels of Creatinine (Cr), Magnesium (Mg), and Blood Urea Nitrogen (BUN)

**Figure 1:**
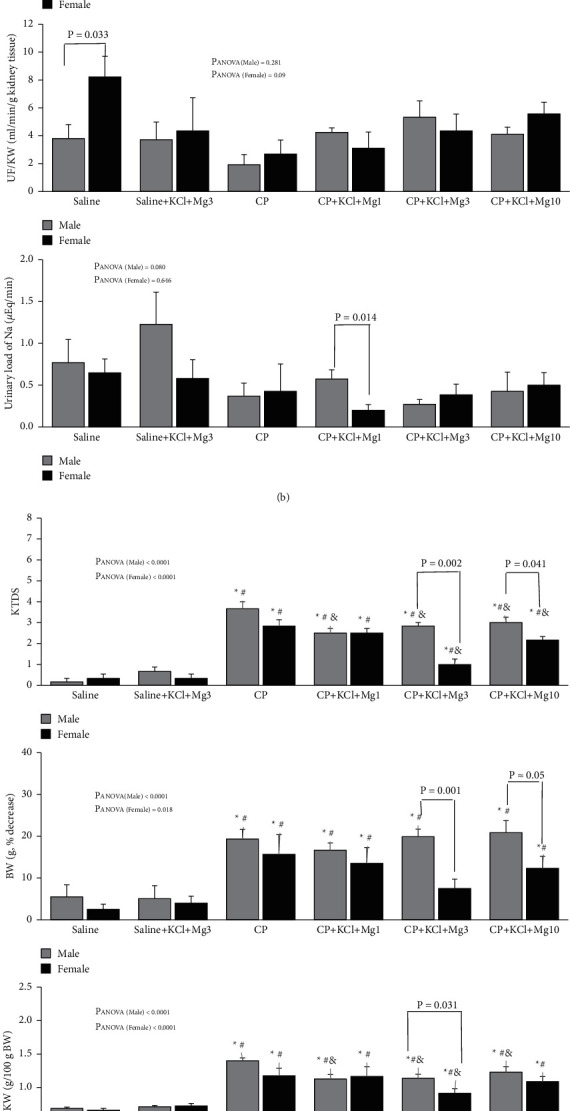
(a) The serum levels of blood urea nitrogen (BUN), creatinine (Cr), and magnesium (Mg), (b) clearance of Cr (ClCr), urine flow (UF), and urinary load of sodium, (c) kidney tissue damage score (KTDS), percentage decrease of body weight (BW), and normalized kidney weight (KW), and (d) the serum levels of malondialdehyde (MDA), nitrite, and lactate dehydrogenase (LDH) in 12 groups of experiments including saline, saline + KCl + Mg3, CP, CP + KCl + MgSO_4_, CP + KCl + Mg1, CP + KCL + Mg3, and CP + KCl + Mg10 (see Table 1 for groups' details). ^∗^, #, and & indicated significant differences from saline, saline + KCl + Mg3, and CP groups, respectively.

The serum levels of BUN and Cr increased significantly (*P* < 0.05) by CP alone in both male and female rats (Figure 1). However, when CP was accompanied with MgSO_4_ supplements (all doses), the serum mean levels of BUN and Cr were reduced in both sexes (Figure 1). The level of Mg was increased by CP alone in both male and female rats (Figure 1). However, the MgSO_4_ supplements accompanied with CP did not alter the serum level of Mg in males, but the mid-dose of MgSO_4_ supplement in the CP + KCl + Mg3 group decreased the serum level of Mg to the normal level in females (Figure 1). Finally, the mid-dose of MgSO_4_ supplement provided significant differences in serum levels of BUN, Cr, and Mg between sexes (BUN: *P*=0.002, Cr: *P*=0.005, and Mg: *P*=0.014).

### 3.2. Creatinine Clearance (ClCr), Urine Flow (UF), and Urinary Load of Na

CP alone or accompanied with supplements reduced ClCr significantly in male groups when compared with control groups (*P* < 0.05). However, the mid- and high doses of MgSO_4_ supplements improved ClCr in females (Figure 1). The significant differences between sexes were also observed in CP + KCl + Mg3 (*P*=0.05) and CP + KCl + Mg10 (*P*=0.032) groups. UF was significantly higher in females than in males in the saline (control) group (*P*=0.033), and CP caused a decrease of UF in females insignificantly when compared with the saline group. No significant differences in UF were observed between CP-alone-treated groups and CP accompanied with supplements treated groups in males and females (Figure 1). In addition, no significant differences in the urinary load of Na were detected between the groups in both males and females. However, there was a significant difference in the urinary load of Na between two sexes in the CP + KCl + Mg1 group (*P*=0.014) (Figure 1).

### 3.3. Body Weight (BW), Kidney Weight (KW), and Kidney Tissue Damage Score (KTDS)

As expected, CP enhanced the KTDS and percentage decrease of BW and KW in males and females compared to the control group significantly (*P* < 0.05). The three different doses of MgSO_4_ supplements reduced the KTDS and KW in males, and the mid- and high doses of MgSO_4_ supplements reduced the KTDS in females (Figure 1). There were also sex differences in the KTDS and BW change in CP accompanied with mid- (*P*_KTDS_ = 0.002 and *P*_BW_ = 0.001) and high (*P*_KTDS_ = 0.041 and *P*_BW_ = 0.05) doses of MgSO_4_-treated groups (Figure 1). The KW in CP accompanied with the mid-dose of MgSO_4_-treated group was significantly different between the sexes (*P*=0.031).

### 3.4. Serum Malondialdehyde (MDA), Nitrite, and Lactate Dehydrogenase (LDH)

The serum level of MDA increased insignificantly in the CP-alone-treated group in males when compared with the saline group. However, in male sex, the serum level of MDA was not attenuated in the CP accompanied with supplement-treated groups (Figure 1(d)). No significant differences were observed in the serum MDA level between the groups in female sex.

The serum level of nitrite in CP-alone-treated rats was significantly different between the sexes (*P*=0.05), but no significant differences were detected between the groups in both male and female rats (Figure 1(d)), and no statistical differences in LDH were detected between the groups in both sexes (Figure 1(d)).

### 3.5. Histological Findings

**Figure 2:**
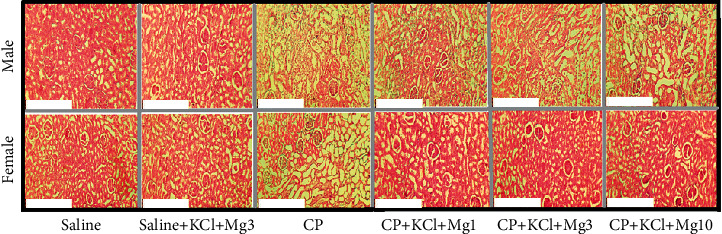
The sample images (×100) of kidney tissues in 12 groups of experiments including saline, saline + KCl + Mg3, CP, CP + KCl + MgSO_4_, CP + KCl + Mg1, CP + KCL + Mg3, and CP + KCl + Mg10 (see Table 1 for groups' details). More damages were seen in the CP-treated group when compared with non-CP-treated groups. The score of damage tissue in the female group treated with CP + KCl + Mg3 was less than the similar treated group of male. The white bar corresponds to 0.25 mm × 1.0 mm.

The sample images of kidney tissues in all the experimental groups are shown in Figure 2. The main important finding was related to female rats of the CP + KCl + Mg3 group which indicated that the mid-dose of MgSO_4_ supplement improved the tissue damage (including hyaline cast, tubular atrophy, necrosis, vacuolization, and debris) in female rats when compared with the CP-alone-treated group (Figure 2).

## 4. Discussion

As a major limiting side effect of CP therapy, it is necessary to manage CPIN to enhance the safety and efficacy of CP during chemotherapy. In addition, developing new prophylactic strategies plays an important role in preventing CPIN. In this study, we evaluated the nephroprotective effect of intravenous administration of MgSO_4_ supplement against CPIN in male and female rats.

Our results indicated that three different doses of MgSO_4_ more and less may prevent CPIN, and the findings are compatible with the results of previous studies [30, 34]. Particularly, coadministration of CP and MgSO_4_ significantly attenuated the elevated serum levels of BUN and Cr induced by the administration of CP.

It is reported that renal organic cation transporter 2 (rOCT2) and renal multidrug and toxin extrusion protein 1 (rMate1) have an important role in modulating the pharmacokinetics of renal platinum [35]. Moreover, under normal conditions, Mg reabsorption takes place primarily in the ascending limb of the loop of Henle (70%) and proximal tubule (15%) [36], and Mg deficiency augments platinum accumulation, while Mg replacement therapy attenuates platinum accumulation induced by Mg deficiency. Also, Mg has antioxidant properties and scavenges oxygen radicals, probably by affecting the rate of spontaneous dismutation of the superoxide ion [37]. It is known that one of the mechanisms of CPIN could be oxidative stress, and Mg may directly relieve the oxidative stress caused by CP administration [29]. Possibly, all of the above have been involved in the reduction process of increased BUN and Cr levels by CP. It has also been reported that CPIN occurred more frequently in male than female rats, being related to the fact that the expression level of OCT2 is higher in males than in females [38].

Mg is the second most common intracellular cation in the body, and it is an active participant in numerous cellular processes, and it is considered as a cofactor for about 300 cellular enzymes [39, 40]. Mg is being filtered at the glomeruli as part of the filtration-reabsorption process, and homeostasis is closely regulated by the kidney [41].

Mg depletion is a side effect of CP which occurs two weeks after CP administration [42]. Unexpectedly, we observed that the Mg serum level increased in all CP-treated groups six days after CP administration possibly due to the reduction of GFR. However, there was no difference in serum Mg levels between the groups which received CP with MgSO_4_ supplements, and the results are compatible with the results of previous studies [32].

In addition, the accumulation of CP in the renal system enhances ROS production and tumor necrosis factor alpha (TNF-*α*) and induces inflammation, oxidative stress, and vascular injury [15, 43]. It is reported that Mg also reduces oxidative stress [44–46]. For example, in renal ischemia-reperfusion injury, the MgSO_4_ supplementation prevents the renal hemodynamic abnormalities such as GFR and RBF reduction via improving endothelial function [47].

ClCr in the CP group was decreased in male and female rats, and this is in agreement with the study [26]. The reduction of UF by CP may be related to a decline in GFR and renal blood flow (RBF) due to renal vascular resistance alteration [14, 48]. However, it is observed that ClCr was improved in the CP + KCl + Mg3 and CP + KCl + Mg10 groups only in female rats, and possibly, the antioxidant and vasodilatory effects of Mg are involved.

Our results showed that weight loss in CP-treated rats is higher than control groups which may be related to disturbance in gastrointestinal or tubular absorption [49–51], as well as loss of the skeletal muscle and apoptosis [52]. The CP-induced weight loss was also confirmed by our previous studies [53–55].

The results of kidney weight and KTDS indicated that intensity of kidney toxicity in the mid-dose of MgSO_4_ supplementation in female rats is lower than male rats. In fact, rOCT2 is Mg dependent, and its expression is higher in male than female rats [38]. These phenomena increase the KTDS in male rats due to the accumulation of CP in the kidney [56]. It was also showed that rOCT2 expression is upregulated by testosterone, but is moderately downregulated by estradiol [57], resulting in greater renal uptake clearance of CP in male than female rats [38]. However, there have been conflicting results regarding sex as a risk factor for CPIN in clinical trials [58, 59].

The serum nitrite level in CP-treated female rats was higher than male rats. The female sex hormone induces the production of nitric oxide (NO) [60]. On the contrary, CP induces endothelial dysfunction and increases the inducible NO synthase (iNOS) level [61]. Therefore, excessive NO reacts with superoxide and produces peroxynitrite which is a potent vasoconstrictor and easily causes oxidative damage to cellular structures [62, 63]. In this study, the serum levels of nitrite in female rats treated with CP alone were more than male rats, probably due to increasing iNOS level and the presence of estrogen. Mg deficiency can also increase plasma NO in rats [64], and NO may enhance the CP toxicity [65]. Our finding is in agreement with others as the serum level of NO metabolite (nitrite) elevated in female rats of the CP-treated group [66]. Previous studies revealed that coadministration of CP and estrogen may promote the severity of toxicity due to estrogen-induced NO production [67].

The findings also showed that CP causes to increase serum levels of MDA in male rats insignificantly. However, coadministration of MgSO_4_ and CP did not attenuate the increased level of MDA induced by CP administration. It seems that the MgSO_4_ supplement is not able to reduce the CP-induced oxidative stress, and more studies are needed to find the exact mechanism.

The current study had some limitations. First, the duration of the animal experiment was designed for six days. However, in order to determine the final effect of intravenous MgSO_4_ supplement on CPIN, more time may be needed to specify the final effects. Second, in order to determine ClCr, 24-hour urine collection is suggested. However, we had 4 hours of urine collection.

## 5. Conclusion

Cointravenous administration of MgSO_4_ with CP ameliorates the incidence and severity of CPIN. Mg supplement attenuates the augmented CP accumulation induced by Mg deficiency, and it works as a cofactor of cellular enzymes, and it is therefore possible that Mg premedication affects the expression and function of renal transporters which is affected by CP. The protective role of MgSO_4_ on CPIN is also gender related. From these results, it appears that the renal protective effect of MgSO_4_ could be dose and gender related, and clinical trial studies need to be designed in patients to find the effective dose of MgSO_4_ supplement against CPIN in different genders.

## Figures and Tables

**Table 1:** The summary of study groups. The duration time of fluid administration before, during, and after CP therapy was 45, 30, and 45 minutes, respectively, using a microinfusion pump.

Group number	Gender	Group name	Treatment		
			Before CP therapy	During CP therapy	After CP therapy
1, 7	M, F	Saline	2 ml of saline	1.5 ml of saline alone	2 ml of saline
2, 8	M, F	Saline + KCl + Mg3	2 ml of fluid containing saline, 0.015 meq/ml of KCl, and 3 mg/ml of MgSO_4_	1.5 ml of saline alone	2 ml of fluid containing saline, 0.015 meq/ml of KCl, and 3 mg/ml of MgSO_4_
3, 9	M, F	CP	—	1.5 ml of saline containing 7.5 mg/kg of CP	—
4, 10	M, F	CP + KCl + Mg1	2 ml of fluid containing saline, 0.015 meq/ml of KCl, and 1 mg/ml of MgSO_4_	1.5 ml of saline containing 7.5 mg/kg of CP	2 ml of fluid containing saline, 0.015 meq/ml of KCl, and 1 mg/ml of MgSO_4_
5, 11	M, F	CP + KCl + Mg3	2 ml of fluid containing saline, 0.015 meq/ml of KCl, and 3 mg/ml of MgSO_4_	1.5 ml of saline containing 7.5 mg/kg of CP	2 ml of fluid containing saline, 0.015 meq/ml of KCl, and 3 mg/ml of MgSO_4_
6, 12	M, F	CP + KCl + Mg10	2 ml of fluid containing saline, 0.015 meq/ml of KCl, and 10 mg/ml of MgSO_4_	1.5 ml of saline containing 7.5 mg/kg of CP	2 ml of fluid containing saline, 0.015 meq/ml of KCl, and 10 mg/ml of MgSO_4_

KCl: potassium chloride; MgSO_4_: magnesium sulfate; M: male; F: female.

## Data Availability

The research data used to support the findings of this study are available from the corresponding author upon request.
